# Upgrading Grape Pomace through *Pleurotus* spp. Cultivation for the Production of Enzymes and Fruiting Bodies

**DOI:** 10.3390/microorganisms7070207

**Published:** 2019-07-21

**Authors:** Aikaterini Papadaki, Vasiliki Kachrimanidou, Seraphim Papanikolaou, Antonios Philippoussis, Panagiota Diamantopoulou

**Affiliations:** 1 Laboratory of Edible Fungi, Institute of Technology of Agricultural Products (ITAP), Hellenic Agricultural Organization—Demeter, 1 Sofokli Venizelou Street, 14123 -Lykovryssi, 14123 Attiki, Greece; 2 Department of Food Science & Human Nutrition, Agricultural University of Athens, Iera Odos 75, 11855 Athens, Greece; 3 Department of Food Science and Technology, Ionian University, 28100 Argostoli, Kefalonia, Greece

**Keywords:** bioconversion, fungi, mushroom, winery side-streams, phenolic compounds, laccase, endoglucanase, fermentation

## Abstract

Grape pomace, a by-product derived from winery industries, was used as fermentation media for the production of added-value products through the cultivation of two *Pleurotus* species. Solid-state (SSF), semiliquid (SLF), and submerged (SmF) fermentations were carried out using grape pomace as substrate. The effect of the different fermentations on the consumption of phenolic compounds, the production of mycelial mass and enzymes was evaluated using *P. ostreatus* and *P. pulmonarius*. The production of fungal biomass and enzymes was influenced by the fermentation mode. The maximum biomass values of ~0.5 g/g were obtained for both *P. pulmonarius* and *P. ostreatus* in SmF. Laccase production was induced in SSF and a maximum activity of 26.247 U/g was determined for *P. ostreatus*, whereas the highest endoglucanase activity (0.93 U/g) was obtained in the SmF of the same fungi. Analysis of phenolic compounds showed that both strains were able to degrade up to 79% of total phenolic content, regardless the culture conditions. Grape pomace was also evaluated as substrate for mushroom production. *P. pulmonarius* recorded the highest yield and biological efficiency of 14.4% and 31.4%, respectively. This study showed that mushroom cultivation could upgrade winery by-products towards the production of valuable food products.

## 1. Introduction

Mushroom cultivation has been widely applied in many regions worldwide owing to its their medicinal and nutritional advantages. The high protein content eliciting all essential amino acids, the low-fat content, the composition in dietary fiber (chitin, hemicellulose, β-glucans, mannans, xylans, and galactans), along with the taste and aroma, constitute some of the unique dietary characteristics of edible mushrooms [1]. Cultivation requirements along with the possibility to utilize agro-industrial renewable resources complements the emerging demands for mushroom cultivation. Regardless the hundreds of mushroom species that can be naturally grow, few of them are commercially cultivated to be consumed by humans, including *Pleurotus* spp., which is among the most cultivated mushrooms [2]. 

*Pleurotus* mushrooms are recognized as a rich source of protein, fiber, carbohydrates, vitamins, and minerals, as well as for its unique flavor. These medicinal mushrooms are well-known for their antitumor, antibiotic, antibacterial, hypocholesterolic, immunomodulation, and prebiotic properties [2,3]. Additionally, their ability to secrete an ample range of enzymes with biotechnological interest has been identified many years ago. More specifically, *Pleurotus* spp. present the ability to secrete extracellular enzymes to degrade lignocellulosic raw materials [4]. Laccase, often know as polyphenol oxidase, and endoglucanase belong to the group of ligninolytic and cellulolytic enzymes, respectively [5,6]. Numerous studies have demonstrated the potential of laccases and cellulases applications in the food and beverage sector [7]. In particular, these enzymes have been applied for the removal of phenolics with the aim to prevent browning, stabilization of fruit juice, wine, and beer, to enhance the structure of gluten during baking, to improve the leavening of bread, for gel formation using sugar beet, and for the treatment of olive oil mill effluents [2,5,8,9,10,11].

Several studies have demonstrated the potential of agro-industrial by-products utilization for *Pleurotus* spp. mushroom cultivation, such as banana stalks, coffee husks, paddy straw, rice straw, wheat straw, cotton waste, peanut shells, and spent mushroom substrate, among others [1,4,12,13]. Wine production constitutes a paramount agricultural and manufacture sector with the annual global production to be estimated at 282 million hectoliters in 2018 [14]. The process of wine manufacture, from the field to the final product, results in the generation of both liquid and solid by-products, including grape stalks, grape pomace, and wine lees. Grape pomace (or grape marc) accounts for approximately 20% of the total weight of initial grapes processed for wine production [15]. It constitutes a lignocellulosic material containing pressed skins, seeds and pulp, including also stems in the case of red vinification process. An annual global capacity of 10.5–13.1 million tons of grape pomace can be projected, considering that 6 L of wine entail 1 kg of grape marc [16]. Hence, various strategies are undertaken to valorize grape pomace as a low-cost material, including ethanol and bioethanol production [17], anaerobic digestion and vermicomposting [16], the extraction of polyphenols [15], animal feed, and soil amendment [16]. Grape pomace has been also evaluated in solid state fermentation, using the fungal strain *Aspergillus awamori* for the production of enzymes, including xylanase and endoglucanase [11].

A recent study was conducted using winery and vineyard waste streams for the cultivation of *Ganoderma lucidum* and *P. ostreatus* mushrooms during solid state fermentation, whereas submerged fermentations were also performed with *G. lucidum*, *L. edodes*, and *P. ostreatus* to assess mycelia growth and protein content [18]. Ongoing research is focusing to elicit bioprocesses to convert grape marc into high-value components (e.g., mushrooms, enzymes, and polysaccharides) via the biotechnological route. Likewise, these compounds can find applications in food processing, and be included again in the food supply chain. Also, in line with the transition to a circular economy, as imposed by European legislations, it is of utmost importance to implement agro-industrial waste and by-products streams to foster sustainable and environmentally benign solutions that will generate diversified end-products that can re-enter the food industry. Hence, the aim of this study was to valorize grape pomace as a substrate for the cultivation of two *Pleurotus* species using diverse fermentation configurations. The production of mycelial mass, laccase and endoglucanase enzymes were evaluated in different fermentation modes, as well as the ability of these strains to grow and produce fruiting bodies in grape pomace.

## 2. Materials and Methods 

### 2.1. Fungal Strains and Inoculum Preparation

The mushroom strains used in the experiments—*Pleurotus ostreatus* AMRL 135 and *Pleurotus pulmonarius* AMRL 177—were obtained from the fungal culture collection of the Laboratory of Edible Fungi /Institute of Technology of Agricultural Products (LEF, ITAP located in Lykovryssi, Attiki, Greece). Potato Dextrose Agar (PDA; Merck, Germany) slants were used to maintain fungal strains at 2 ± 0.1 °C. Before each experiment fungal strains were reproduced in PDA Petri dishes by incubation at 26 ± 1 °C for 7 days.

Inoculum was prepared in two sequential liquid precultures using initially a synthetic glucose-based medium (pH 6.1) consisting of glucose, 10 g/L; yeast extract, 1.5 g/L; KH_2_PO_4_, 7 g/L; K_2_HPO_4_, 2.5 g/L; MgSO_4_·7H_2_O, 1.5 g/L; (NH_4_)SO_4_, 1.3 g/L; CaCO_3_, 0.2 g/L; CaCl_2_·2H_2_O, 0.15 g/L; FeCl_3_·6H_2_O, 0.15 g/L, MnSO_4_·H_2_O, 0.04 g/L; ZnSO_4_·7H_2_O, 0.02 g/L; and thereafter the grape pomace hydrolysate (as described in Section 2.5.1). Erlenmeyer flasks of 500 mL filled with 150 mL of synthetic media were sterilized for 20 min at 121 ± 1 °C and after cooling, inoculation with two PDA agar disks (7 mm diameter) was performed. Incubation was carried out at 26 ± 1 °C for 10 days using an agitation rate of 140 rpm (orbital shaker, ZHICHENG ZHWY 211C, China). Then preculture was aseptically homogenized and used as inoculum (10%, *v/v*) for the second preculture which contained the same synthetic medium but with 5 g/L glucose and 0.75 g/L yeast extract. Incubation was followed for 5 days at 26 ± 1 °C and 140 rpm.

### 2.2. Raw Materials and Fermentation Media

Grape pomace was obtained from the red wine making process of “Agiorgitico” grape variety, performed in Laboratory of Enology /Institute of Technology of Agricultural Products (LE, ITAP located in Lykovryssi, Attiki, Greece). Analysis of the chemical profile of grape pomace [19,20] showed that the main constituents were (expressed in *w/w* dry basis): crude fibers (22.0%), soluble sugars (20.0%), total Kjeldahl nitrogen (14.2%), and ash (10.2%). Samples were collected after mechanical pressing, dried at 80 ± 0.1 °C and mechanically grinded to obtain particle sizes< 0.8 mm. Grape pomace was employed as the growth medium for all fermentation modes (SSF, SmF, and SLF). 

### 2.3. SSF, SmF and SLF Conditions

Erlenmeyer flasks containing 4 g of grape pomace (dry basis) were autoclaved for 20 min at 121 ± 1 °C. In the case of SmF and SLF, distilled sterilized water was added to achieve final grape pomace concentrations of 0.04 g/mL and 0.2 g/mL, respectively [21,22]. Substrates were inoculated with 10 mL of liquid preculture of each strain, followed by incubation at 26 ± 1 °C in an orbital shaker (140 rpm) (MPM M301-OR, Italy) for 20 days. Three replicates were employed for each strain and each fermentation process tested. Immediately after sample collection, the extraction of phenolic compounds and analysis of moisture content were performed, whereas the remaining sample was freeze-dried (Heto LyoLab 3000 freeze-dryer, Heto-Holten Als, Denmark) and stored for further analysis of glucosamine content and enzymatic activities.

### 2.4. Cultivation Conditions for Fruiting Bodies Production

Grape pomace was evaluated for fruiting bodies production using both *Pleurotus* strains. Grape pomace was initially soaked in water for 12 h, and afterwards the surplus water was drained off 5% wheat bran, 5% soybean flour, and 1% CaCO_3_ were added as supplements. Glass containers of 600 mL volume were filled with the substrate and autoclaved for 20 min at 121 ± 1 °C. The moisture content of the medium after sterilization was 55% and pH was 6.5. Inoculation was carried out by adding liquid preculture into the central vertical axis of the jar, followed by incubation in growth chambers at 26 ± 1 °C until complete colonization of the substrate. Subsequently, fructification was induced by adjusting the environmental conditions. More specifically, light intensity was set at 200 lux (12 h/day, fluorescent lamps), air exchange rates were regulated to maintain CO_2_ level< 1200 ppm, relative air humidity was adjusted at 90%, and temperature was set at 18 ± 1 °C [4].

The mature fruiting bodies were harvested daily, counted and weighted. Additionally, the parameters earliness, which is the days elapsed between the day of inoculation and the day of the first harvest; biological efficiency (BE), which is the percentage yield of fresh mushrooms harvested per g of dry substrate; total yield, which is the percentage yield of fresh mushrooms harvested per g of substrate; and the average fresh weight of fruiting bodies, were evaluated [4]. Results represent the average value of eight replicates.

### 2.5. Analytical Methods

#### 2.5.1. Hydrolysis of Grape Pomace and Biomass Production

Acid hydrolysis of grape pomace was carried out to obtain a fermentation media, which was used for inoculum preparation (as described in Section 2.1) and for fungal biomass production. The biomass obtained through SmF using the hydrolysate, was correlated with its glucosamine content (Section 2.5.2. Specifically, a known amount of grape pomace (dry basis) was mixed with ethanol (95%), in a proportion 1:5 (*w/v*), and heated up to 100 °C for 5 min. Subsequently, the solution was left to cool at room temperature, filtered with Whatman paper (Whatman No. 3), and extracted two more times following the same procedure. The last extraction was carried out with absolute ethanol, and the sediment was left to dry for 12 h [23,24]. Sample was then mixed with H_2_SO_4_ (72%) (dry solids:acid 1:12.5, *w/v*), and the mixture was left for 3 h at 20 °C. After that, distilled water was added to achieve a solution of 1 M H_2_SO_4_, and acid hydrolysis was performed for 2.5 h at 100 °C. The final hydrolysate was obtained after filtration and neutralization using 1 M KOH [20,24,25]. 

Then hydrolysate was supplemented with 4 g/L glucose, 0.75 g/L yeast extract and the other supplements, as mentioned in Section 2.1, and biomass was produced through fermentation of each strain at 26 ± 1 °C for 6 days under agitation (140 rpm). Biomass was separated from fermentation samples through filtration (Whatman No 1) and washed three times with distilled water. The clear broth filtrate was collected and stored at −20 ± 1 °C until further analysis of sugar consumption, whereas fungal biomass was transferred in preweighed McCartney bottles and dried at 60 ± 1 °C until a constant weight was achieved. Sugar concentration was determined in broth filtrate of the preculture using the 3,5-dinitro-2-hydroxy-benzoic acid (DNS) method for reducing sugars [26] and phenol and the sulfuric acid method for the total sugars [27].

#### 2.5.2. Determination of Glucosamine Content 

A glucosamine standard curve was obtained using increasing concentrations of N-acetyl-D-glucosamine (Sigma-Aldrich). Subsequently, the biomass of each *Pleurotus* strain, which was obtained through SmF in grape pomace hydrolysate, was hydrolyzed and glucosamine content was determined. The correlation of known amounts of biomass with glucosamine content resulted in the linear regression equations shown in Table 1.

#### 2.5.3. Indirect Estimation of Biomass in Fermentations

The glucosamine present in the fungal cell wall was used to monitor fungal biomass in all fermentations. The method of chitin hydrolysis into N-acetylglucosamine and the determination of glucosamine content have been previously described [28]. The same protocol was followed using unfermented medium as blank. Results were expressed as g fungal biomass per g of dry substrate.

#### 2.5.4. Crude enzyme extraction and determination of enzyme activities

Approximately 2 g of lyophilized colonized substrate was mixed with 20 mL sodium acetate buffer (0.05 M, pH = 5.0) and agitated (100 rpm) for 1 h at room temperature. The crude extracts were recovered by filtration (Whatman No2, England) followed by centrifugation (10,000× *g*, 15 min, 4 ± 0.1 °C) (Hettich Micro22R, Hettich, Germany). Clear supernatants were stored at −20 ± 1 °C for further analysis of endoglucanase and laccase activities according to the method described by Philippoussis et al. [29]. One unit of endoglucanase was defined as the amount of enzyme producing 1 μmol of reducing sugar (glucose equivalent) in one minute, under the conditions assayed. The standard curve was obtained with glucose for CMC. Laccase was determined using syringaldazine as substrate. One unit of laccase was defined as the amount of enzyme required to produce a change in absorbance of 0.001 per minute, under the conditions assayed. At least triplicates were used for the determination of each enzymatic activity which were expressed as U/g of dry substrate.

#### 2.5.5. Determination of Phenolic Compounds

Phenolic compounds were determined using the Folin–Ciocalteu method as described by Puoci et al. [30]. Briefly, to extract the phenolic compounds, 5 g of fresh colonized substrate was mixed with 20 mL of methanol and sonicated for 1 h, followed by filtration. The filtrate was collected and the same extraction process (30 min sonication) was repeated twice. Filtrates were combined and subsequently the solvent was vacuum evaporated at 40 ± 1 °C. The extracted phenolic compounds were resuspended in 15 mL of methanol and stored at −20 ± 1 °C until further analysis. For the photometric method, 10.8 mL of distilled water were mixed with 0.2 mL extracted sample, 8 mL of Na_2_CO_3_ solution (7.5%, *w/v*) and 1 mL of Folin–Ciocalteu reagent. The samples were left to settle for 2 h allowing the color to develop and the absorbance was measured at 760 nm. Unfermented substrate of each fermentation was utilized to evaluate the initial total phenolic content (TPC), which was expressed as g of gallic acid equivalents per g of dry substrate, using a standard curve. 

### 2.6. Statistical Analysis

The statistical differences for biomass, enzymes production and phenolic compounds reduction were estimated by analysis of variance (ANOVA). Whenever ANOVA indicated a significant difference between variables at a significance level of 5% (*p* < 0.05) the Tukey’s HSD (honest significant difference) test was carried out using the Excel software.

## 3. Results and Discussion

### 3.1. Biomass Production 

Biomass production was indirectly estimated through the determination of glucosamine content. The equations of Table 1 were used to convert glucosamine to biomass. This method has been widely applied to determine biomass production during fermentations of several mushrooms, including *Lentinula edodes*, *Pleurotus* spp., *Ganoderma* spp., and *Morchella* spp. [12,28,29]. The glucosamine content of *Pleurotus* spp. strains was ranged from 18.5 to 20.3 mg/g of dry biomass, which was significantly higher than previous studies (3.6 mg/g) for *P. ostreatus* grown on groundnut shells [31]. Generally, glucosamine content of mycelial mass depends on medium, fungal species and culture conditions [28,29].

Table 2 illustrates that the highest biomass concentrations of 0.50 g/g and 0.54 g/g were obtained in SmF for *P. ostreatus* and *P. pulmonarius*, respectively, at the 20^th^ day of the fermentation. The results indicated that grape pomace supports the growth of *Pleurotus* spp., with *P. pulmonarius* producing higher biomass concentrations in all fermentations than *P. ostreatus*. To the best of our knowledge, there are no literature cited results reporting biomass production of mushrooms using grape pomace, whereas it has been indicated in other agro-industrial substrates. Economou et al. [12] mentioned the highest biomass production of around 0.13-0.14 g/g for *P. ostreatus* and *P. pulmonarius* using spent mushroom substrate in SSF. Other mushrooms, such as *L. edodes* presented similar to this study biomass production (up to 0.46 g/g) during SSF on bean stalks [29].

### 3.2. Consumption of Phenolic Compounds

**Figure 1:**
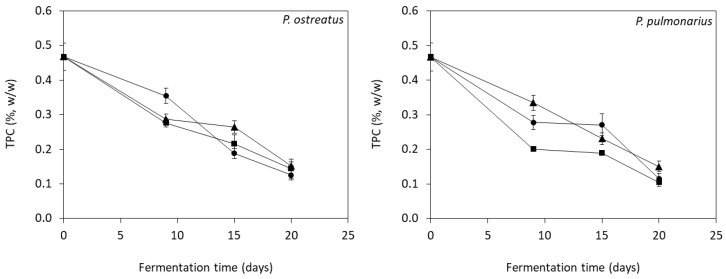
Total phenolic content (TPC) of the colonized substrate during solid-state (SSF, ●), submerged (SmF, ▲) and semiliquid (SLF, ■) fermentations of *P. ostreatus* and *P. pulmonarius* using grape pomace as substrate.

TPC of colonized grape pomace was evaluated during the fermentations and the results are shown in Figure 1. In all fermentation modes, both *Pleurotus* strains were able to consume significant amount (*p* < 0.05) of the phenolic compounds as compared with the initial TPC in unfermented grape pomace, which was 0.47 ± 0.04% (*w/w*). Particularly, the highest TPC reduction of 79% was determined in SLF of *P. pulmonarius*, followed by 74% in SSF, whereas the lowest TPC reduction of 68% was noticed in SmF. The same behavior was observed for *P. ostreatus*, which presented the highest TPC reduction in SSF and SLF (72% and 70%, respectively) and the lowest one in SmF (68%). It is noteworthy to mention that biomass was steadily increased with decreasing TPC. Specifically, biomass production in each fermentation was correlated with the respective values of TPC and negative relations with high regression coefficient (R^2^ ranged from 0.63 to 0.98) were revealed.

Sanchez et al. [32] reported lower phenolic removal by *P. ostreatus* and *P. pulmonarius* (18.4% and 9.2%, respectively) cultivated in grape pomace. Gaitán-Hernández et al. [33] demonstrated a TPC reduction of 71.4% by *L. edodes* in viticulture residues. The phenolic removal has been also investigated in other phenolic-rich substrates. Many studies have been focused on the utilization of olive oil mill wastewater as fermentation medium by *L. edodes*, *P. ostreatus*, and *P. pulmonarius* reporting a final phenolic removal up to 76% [34,35]. The highest phenolic removal of ~80% has been determined during SSF of *P. ostreatus* in coffee pulp [36].

### 3.3. Laccase Production 

**Figure 2:**
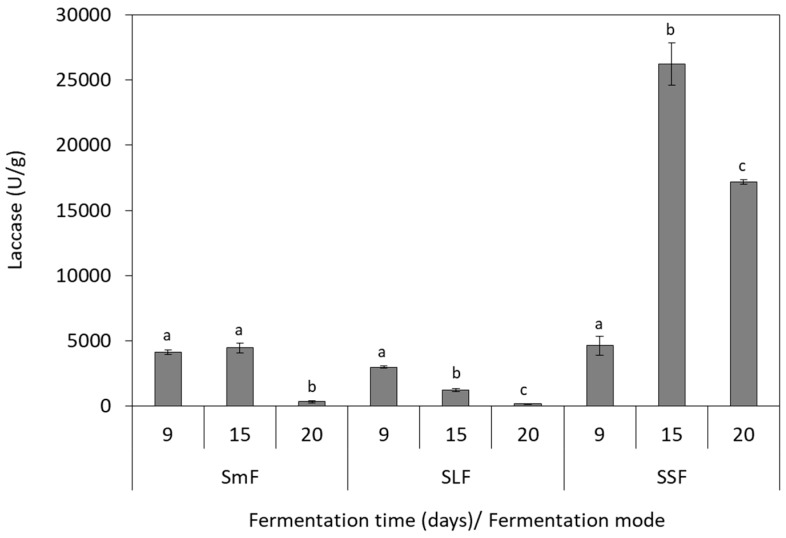
Production of laccase by *P. ostreatus* during solid-state (SSF), submerged (SmF), and semiliquid (SLF) fermentations using grape pomace as substrate. Different letters indicate significant differences (*p* < 0.05) between fermentation days for each fermentation mode.

**Figure 3:**
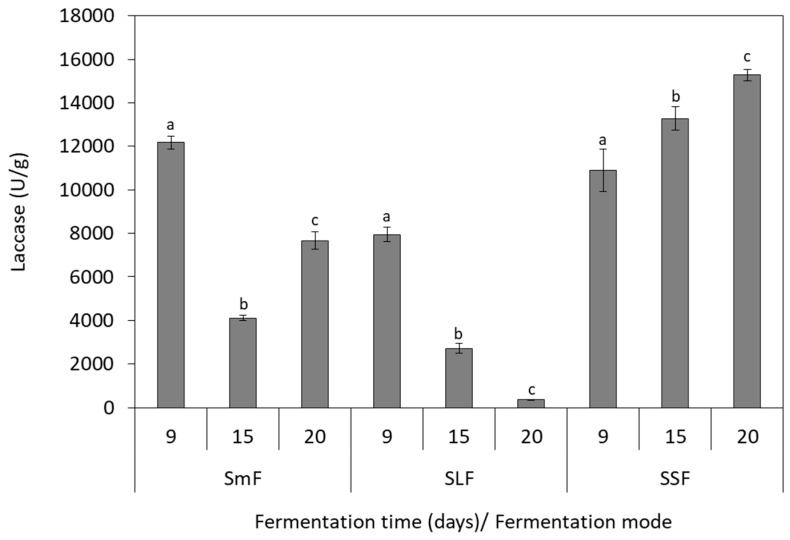
Production of laccase by *P. pulmonarius* during solid-state (SSF), submerged (SmF), and semiliquid (SLF) fermentations using grape pomace as substrate. Different letters indicate significant differences (*p* < 0.05) between fermentation days for each fermentation mode.

Laccase activity was influenced by the fungal species and the fermentation mode, as depicted in Figure 2 and Figure 3. The highest values of 26,247.0 U/g (15th day) and 15,273.0 U/g (20th day) were determined in SSF for *P. ostreatus* and *P. pulmonarius*, respectively. The lowest enzyme activities were observed in SLF, indicating that this fermentation mode cannot support the efficient laccase production for both *Pleurotus* species. *P. ostreatus* presented low laccase activity also in SmF, with the highest value of 4447.0 U/g produced at 15th day of the fermentation (Figure 2). Laccase production from *P. pulmonarius* illustrated different pattern in SmF, as compared to *P. ostreatus*. In this case, the highest laccase activity of 12,174.0 U/g was determined at the 9th fermentation day (Figure 3). It was also noticed that laccase production and TPC reduction were positively correlated in SSF, which is attributed to the fact that laccase is involved in degradation of the phenolic compounds. This finding is in agreement with previous studies evaluating phenolic compounds consumption by other mushrooms using olive oil mill wastewater as substrate [34,35].

Results showed that SSF was the best fermentation mode for laccase production. Many researchers have indicated that low laccase production was observed when the moisture content of the substrate was increased, due to the lowest oxygen transfer [36,37]. This can be explained by the fact that laccases act on phenols by using oxygen as the electron acceptor [35]. Thus, the highest laccase production observed in this study during SSF could be attributed to better oxygen transfer as compared to the other fermentation modes. Moreover, Stajić et al. [38] reported that laccase production is highly dependent on *Pleurotus* spp. and fermentation mode (SSF or SmF). Particularly, *P. eryngii* presented the highest laccase activity in SmF using mandarin peels, whereas *P. ostreatus* cultivation resulted in the lowest enzyme activity under these conditions. *P. ostreatus* and *P. pulmonarius* showed the highest laccase activity in SSF of grapevine sawdust in comparison to the other substrates [38]. Sadh et al. [1] reported that SSF processes are often advantageous over other fermentation modes owing to higher enzyme production and higher yields, low operational cost and lower risk of contamination. 

In this study, *P. ostreatus* was able to produce higher quantities of laccase than *P. pulmonarius* in grape pomace. However, the substrate is an important parameter that affects enzyme production. For instance, Economou et al. [12] demonstrated higher laccase production from *P. pulmonarius* (44,363.2 U/g) in comparison with *P. ostreatus* (12,751.7 U/g) during SSF using spent mushroom substrate. The production of oxidative enzymes from *Pleurotus* strains have been extensively studied on various substrates, such as wheat bran, coffee pulp, olive oil mill wastewater, mandarin peels and spent mushroom substrate [12,37,39,40,41,42]. Similar laccase production (20,000.00 U/g) has been mentioned for *P. pulmonarius* during SSF on wheat straw [37]. However, there are few studies investigating the production of laccase on winery substrates. Elisashvili et al. [22] studied the SmF of *P. ostreatus* in grape pomace, which resulted in low laccase production of 750 U/L. Enhancement of laccase production by *Pleurotus* species has been achieved by the supplementation of the medium with Cu^2+^ and Mn^2+^ [43,44].

### 3.4. Endoglucanase Production

**Figure 4:**
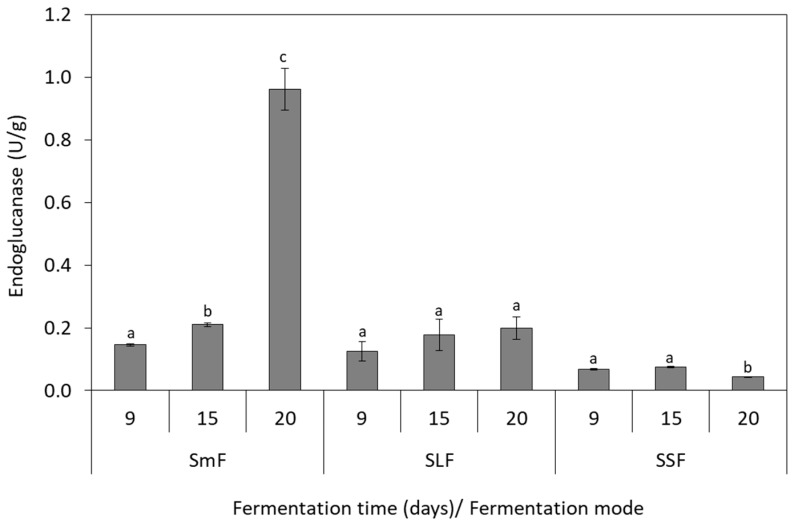
Production of endoglucanase by *P. ostreatus* during solid-state (SSF), submerged (SmF), and semiliquid (SLF) fermentations using grape pomace as substrate. Different letters indicate significant differences (*p* < 0.05) between fermentation days for each fermentation mode.

**Figure 5:**
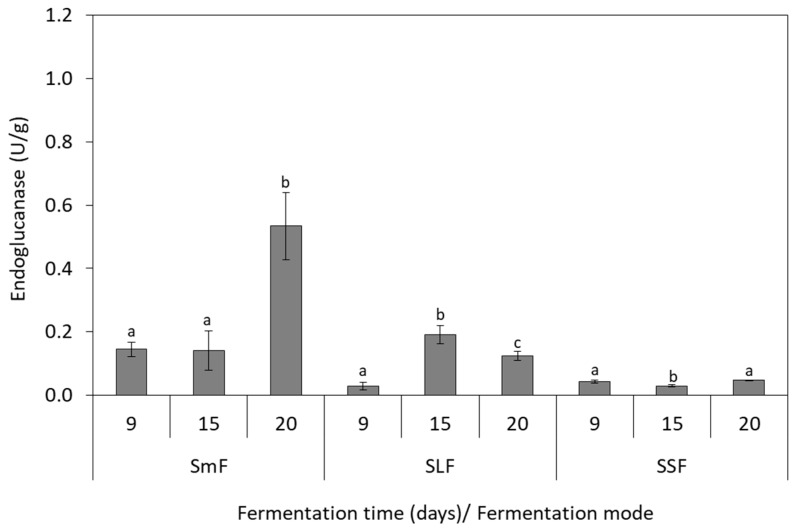
Production of endoglucanase by *P. pulmonarius* during solid-state (SSF), submerged (SmF), and semiliquid (SLF) fermentations using grape pomace as substrate. Different letters indicate significant differences (*p* < 0.05) between fermentation days for each fermentation mode.

Endoglucanase activity was induced in SmF as the highest values of 0.93 U/g and 0.56 U/g were obtained at 20th day for *P. ostreatus* (Figure 4) and *P. pulmonarius* (Figure 5), respectively. Lower endoglunase activity was determined in SLF (~0.2 U/g) and SSF (0.05–0.07 U/g) for both strains. Biomass production and endoglucanase activity presented a positive correlation in SmF, which was indicated with a high regression coefficient for *P. ostreatus* (R^2^ = 1) and *P. pulmonarius* (R^2^ = 0.93).

The results of this study are supported by the findings of Elisashvili et al. [22,45], which indicated that the production of hydrolytic enzymes, such as endoglucanase, was enhanced in SmF as compared to SSF. Few studies have been reported endoglucanase production by *Pleurotus* species in grape pomace (8 U/mL) [45] and viticulture residues (0.28 U/g) [46]. Philippoussis et al. [29] reported endoglucanase activity of 0.97 U/g in wheat straw and 1.71 U/g in bean stalks for *L. edodes* and *G. adsperum*, respectively. An important parameter that affects cellulase activity is the heating pretreatment of the substrate. Karpe et al. [47] demonstrated that autoclaved winery by-products contained higher quantity of free sugars than non-autoclaved, which may inhibit cellulase production in some fungal strains.

### 3.5. Evaluation of Grape Pomace for Fruiting Bodies Production

SSF of *P. ostreatus* and *P. pulmonarius* were carried out in controlled environmental conditions, in order to evaluate their fruiting body production efficiency using grape pomace as substrate. The results of Table 3 revealed differences regarding fruiting body formation by the two *Pleurotus* species. Grape pomace favored the production of *P. pulmonarius* mushrooms presenting three flushes. Noteworthy, grape pomace supported less flushes (only one) in the case of *P. ostreatus*. The cultivation of *P. pulmonarius* exhibited higher yield and BE with shorter earliness period than *P. ostreatus*. The superiority of *P. pulmonarius* over *P. ostreatus* has been also highlighted in cotton waste [48]. Sánchez et al. [32] found slightly higher BE (37–40%) when *P. ostreatus* and *P. pulmonarius* were cultivated in grape pomace. However, BE enhanced using viticulture residues (58–78%).

Generally, *Pleurotus* mushrooms present high BE in straw-based substrates rather than phenolic-rich substrates. Specifically, *P. ostreatus* cultivation on straw substrate supplemented with 30% olive cake showed a BE of 79.9% [49]. Contrarily, a substrate consisting of 90% olive cake had lower BE (26%) [49]. However, the mushrooms produced from the olive cake had higher protein content (35%) than mushrooms produced from wheat straw substrate (26.6%) [49]. This indicates that regardless the low yield achieved with phenolic-rich substrates, the nutritional value of produced mushrooms may be enhanced. Thus, further studies focusing on the optimization of substrates composition could lead to the efficient utilization of winery by-products to produce mushrooms with high nutritional value. 

## 4. Conclusions

The bioconversion of grape pomace for the production of enzymes and fruiting bodies from *Pleurotus* spp. was investigated. This is the first study reporting the effect of different fermentation modes on biomass and enzyme production by *Pleurotus* species. Significant amounts of the initial phenolic content of grape pomace was consumed by *P. ostreatus* and *P. pulmonarius*, which was positively correlated with the biomass production. Higher laccase and endoglucanase activities were achieved by *P. ostreatus*. Specifically, laccase activity was induced in SSF, whereas endoglucanase was reached its maximum activity in SmF. Conclusively, enzyme production was affected by the fermentation mode and *Pleurotus* species. Satisfactory mushroom yield and biological efficiency were observed in the case of *P. pulmonarius,* proving that grape pomace is an alternative substrate for mushroom cultivation. Any further research could be focus on the optimization of the heating pretreatments of grape pomace aiming to higher enzyme activities and mushroom yields [47].

## Figures and Tables

**Table 1:** Linear regression equations of glucosamine (mg) and mycelial biomass (g) of *Pleurotus* strains grown on grape pomace hydrolysate.

*Pleurotus* spp.	Biomass g (y)/glucosamine mg (x)	R^2^
*P. ostreatus*	y = 0.0529x − 0.0735	0.99
*P. pulmonarius*	y = 0.0579x − 0.0715	0.99

**Table 2:** Biomass production (g/g of dry substrate) during solid-state (SSF), submerged (SmF) and semiliquid (SLF) fermentations of *P. ostreatus* and *P. pulmonarius* using grape pomace as substrate.

Time (Days)	*P. ostreatus*			*P. pulmonarius*		
	SSF	SmF	SLF	SSF	SmF	SLF
9	0.31 ± 0.01 ^a^	0.38 ± 0.01 ^a^	0.36 ± 0.01 ^a^	0.34 ± 0.01 ^a^	0.44 ± 0.01 ^a^	0.40 ± 0.01 ^a^
15	0.32 ± 0.02 ^a^	0.40 ± 0.01 ^a^	0.36 ± 0.01 ^a^	0.37 ± 0.01 ^b^	0.46 ± 0.00 ^b^	0.40 ± 0.01 ^a^
20	0.42 ± 0.01 ^b^	0.50 ± 0.02 ^b^	0.43 ± 0.01 ^b^	0.40 ± 0.01 ^c^	0.54 ± 0.01 ^c^	0.45 ± 0.02 ^b^

^a,b,c^ Different letters indicate significant differences for each parameter within the same column (*p* < 0.05).

**Table 3:** Mushrooms yields and biological efficiency (BE) of *P. ostreatus* and *P. pulmonarius* using grape pomace as substrate.

*Pleurotus* spp.	Flushes	Earliness (Days)	Mushroom Number	Average Fresh Weight (g)	Total Yield (%)	BE (%)
*P. ostreatus*	1	42	162 ± 3.7	25.9 ± 2.3	7.4	16.2
*P. pulmonarius*	1	35	176 ± 9.5	24.0 ± 1.8	14.4	31.4
	2	45	43 ± 3.1	14.4 ± 1.4		
	3	55	12 ± 2.6	11.9 ± 1.5		
